# Use of the superior mesenteric artery as an arterial interposition graft in horseshoe kidney transplantation: a case report

**DOI:** 10.1093/jscr/rjag884

**Published:** 2026-10-04

**Authors:** Abigail W Cheng, Karen Bach, Marianne Estrada, Evan Childers, Sunil Patel

**Affiliations:** Department of Surgery, Kirk Kerkorian School of Medicine at University of Nevada, 1701 W Charleston Blvd #400, Las Vegas, NV 89106, United States; Department of Plastic and Reconstructive Surgery, Yale School of Medicine, 800 Howard Ave Ste 4th Floor, 800 Howard Ave Ste 4th Floor, New Haven, CT 06519, United States; Department of Medicine, Kirk Kerkorian School of Medicine at the University of Nevada, 625 Shadow Lane, Las Vegas, NV 89106, United States; Department of Plastic Surgery, Kirk Kerkorian School of Medicine at University of Nevada, 1701 W Charleston Blvd #400, Las Vegas, Las Vegas, NV 89106, United States; Center for Transplantation, University Medical Center Southern Nevada, 901 S Rancho Lane #250, 901 S Rancho Lane #250, Las Vegas, NV 89106, United States

**Keywords:** horseshoe kidney, kidney transplantation, arterial reconstruction, interposition graft, superior mesenteric artery

## Abstract

Vascular reconstruction in kidney transplantation remains a technical challenge, especially in cases involving horseshoe kidneys, which often have anomalous vascular and ureteral anatomy. Proper selection of reconstruction technique and vessel graft source is crucial for long-term organ graft survival. This case report describes the successful use of a donor superior mesenteric artery segment as an interposition graft between a compromised donor renal artery and recipient external iliac artery in a horseshoe kidney transplant. To the best of our knowledge, this is the first reported case where the superior mesenteric artery was successfully utilized as an interposition arterial graft in renal transplantation.

## Introduction

End-stage renal disease (ESRD) affects more than 808 000 people in the United States with a mortality rate of 187.7 per 1000 person-years for those receiving hemodialysis [1]. Kidney transplantation remains the definitive treatment for ESRD, reducing mortality up to 74.3 per 1000 person-years [1]. However, complications can arise due to vascular damage during explantation and back-table preparation, with vascular reconstruction remaining a paramount issue. When vascular damage occurs in conjunction with complex kidney anatomy, the choice of vascular graft and anastomosis technique can be limited.

Horseshoe kidneys have an incidence of 1 in 800 people and are often associated with vascular and ureteral anomalies, making them less desirable for transplantation [2]. Here, we present a case involving a horseshoe kidney transplant and the use of the superior mesenteric artery (SMA) as an arterial interposition graft for arterial reconstruction.

## Case report

A 49-year-old male with ESRD secondary to polycystic kidney disease on hemodialysis was admitted for a deceased donor kidney transplant after being on the waiting list for 3 years. The donor kidney was a left horseshoe kidney that had been divided at the isthmus and oversewing the renal parenchyma. The left kidney had one main artery supplying the upper and middle poles and a smaller artery originating from the lower end of the aorta supplying the lower pole.

During back-table preparation, a dissection was noted in the wall of the main renal artery, ~2 cm from its origin, likely due to poor flushing of the kidney during organ recovery. The donor SMA was evaluated and was utilized as an interposition graft to provide adequate length for anastomosis to the recipient external iliac artery. The SMA graft was attached to the left main renal artery in an end-to-end anastomosis with 7–0 Prolene suture. The kidney was then flushed with University of Wisconsin solution, with good flow and no evidence of any leak.

The kidney was transplanted into the right iliac fossa using standard technique. The SMA graft was anastomosed end-to-side to the external iliac artery with 5–0 Prolene suture after the renal vein was anastomosed to the external iliac vein. A distal arteriotomy was then created on the external iliac artery, to which the lower pole renal artery patch was anastomosed with 6–0 Prolene suture. During reperfusion, the kidney appeared uniformly pink with mild cyanosis at the upper and middle poles, likely secondary to reperfusion injury, which improved with papaverine administration. Postoperative Doppler ultrasound confirmed a good pulse of the main renal artery as well as throughout the kidney. Renal ischemia time was ~30 h. The remainder of the transplantation was performed in the standard fashion without complications.

Postoperatively, renal ultrasound demonstrated patent renal artery and vein, with resistive indices of 0.61, 0.65, and 0.60 for the upper, middle, and lower poles, respectively (Fig. 1). The patient experienced delayed graft function and elevated creatinine levels up to 10.04 mg/dL. Therefore, renal scintigraphy was performed on post-operative day four, which showed good blood flow to all three poles of the transplanted kidney but with poor washout, indicative of delayed graft function (Fig. 2). Given the SMA graft showed good patency and function, no additional anticoagulants outside of the usual regimen were given. The patient required hemodialysis on postoperative days four and six and nine, and was discharged on postoperative day six.

**Figure 1:**
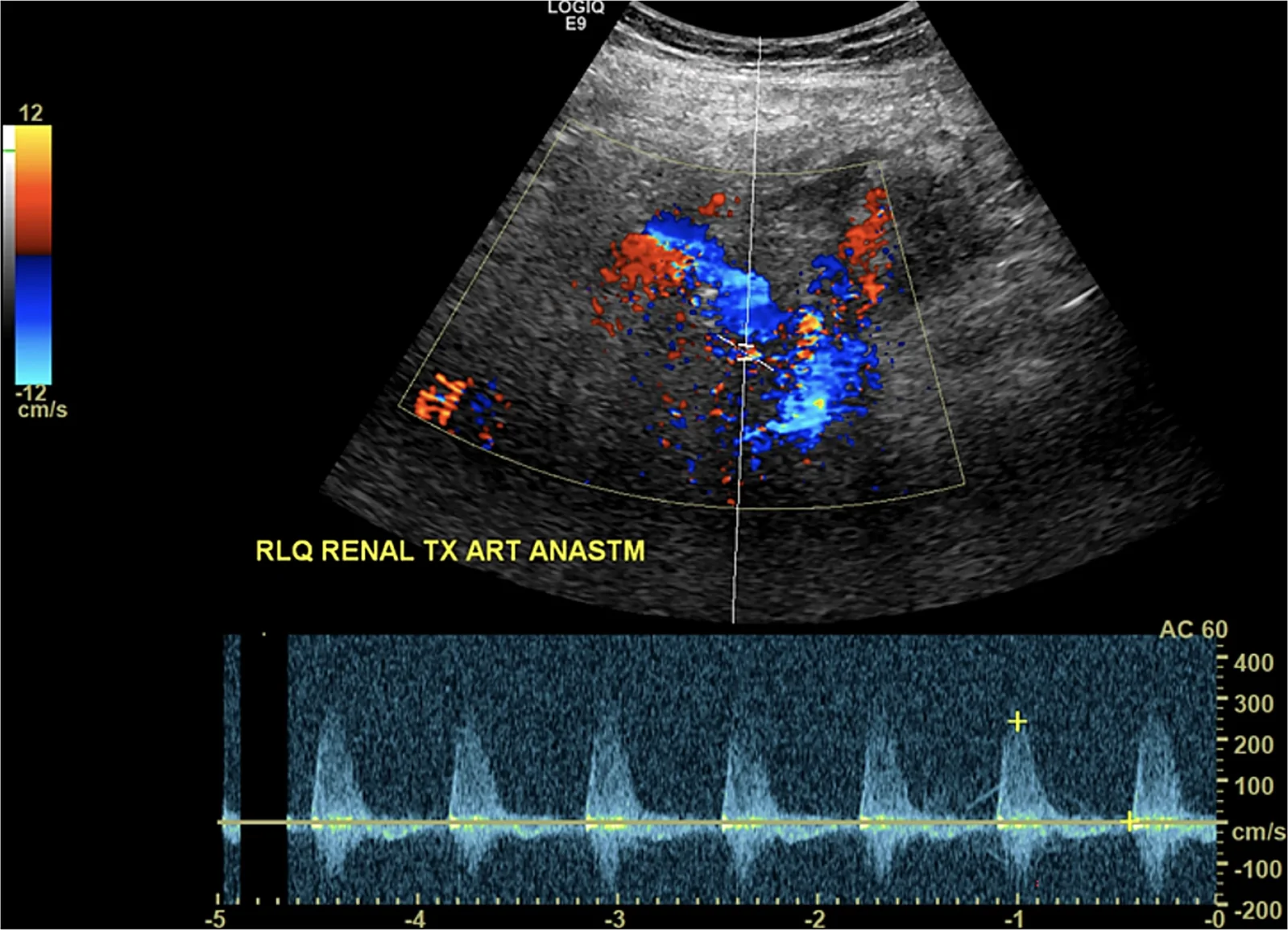
Postoperative renal transplant ultrasound demonstrating patent renal arterial anastomosis, with resistive indices of 0.61, 0.65, and 0.60 at the upper, middle, and lower poles, respectively.

**Figure 2:**
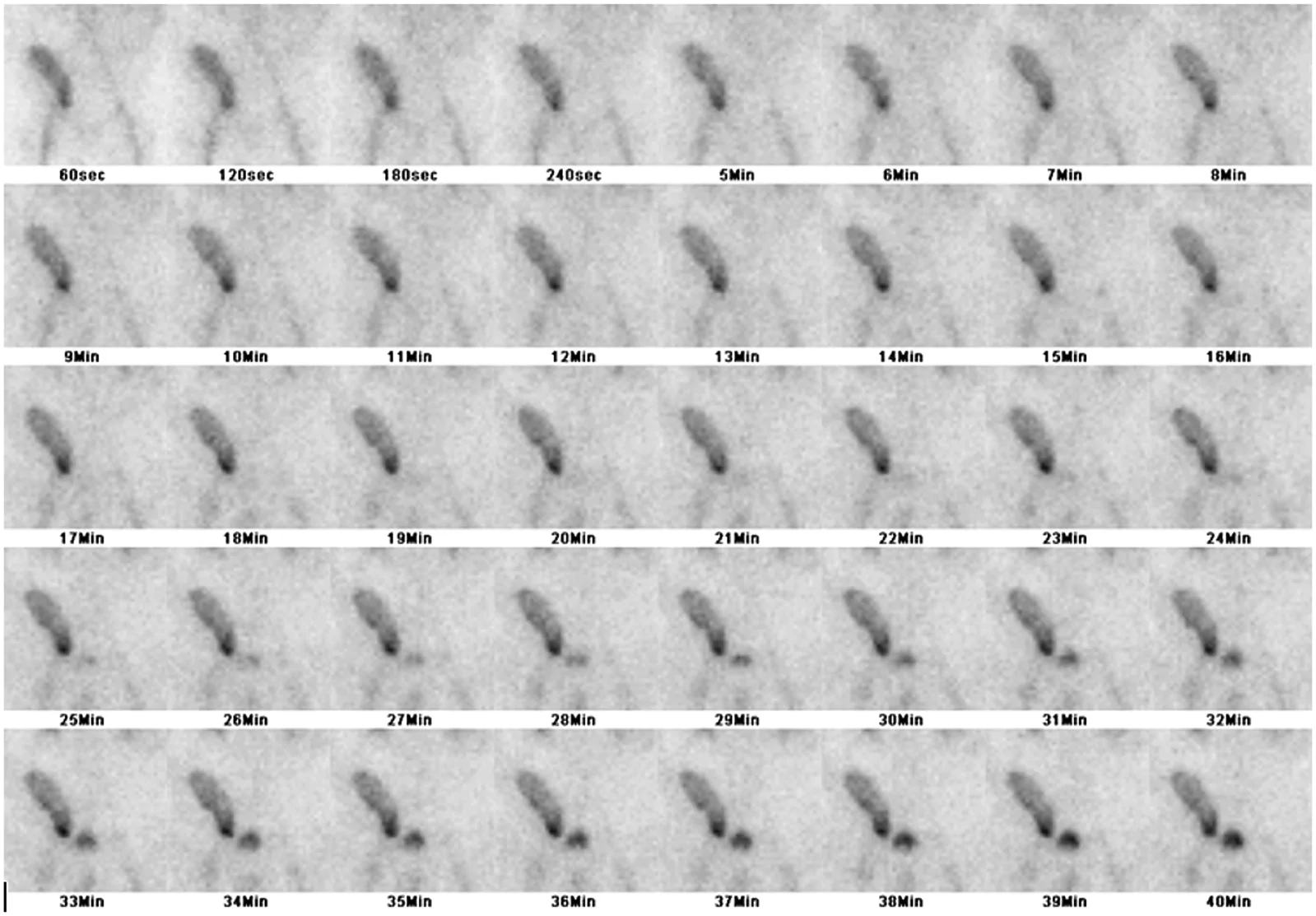
Postoperative day four renal scintigraphy demonstrating good blood flow to all three poles of the transplanted kidney with poor washout, consistent with delayed graft function.

At 12-months post-transplant, the patient achieved stable renal function, with creatinine of 1.68 mg/dl, blood urea nitrogen of 19, and potassium of 4.0.

This case report was reviewed by the University Medical Center Southern Nevada Institutional Review Board (IRB) and deemed exempt from IRB review.

## Discussion

In this case, the donor kidney’s main renal artery had an intimal tear that likely occurred due to high-pressure flushing during organ recovery. Without arterial reconstruction, the donor kidney would have had insufficient arterial length for proper anastomosis and not been feasible for transplantation.

Vessel diameter and composition are important variables to consider in graft selection for vascular reconstruction for adequate blood flow. Although the saphenous vein, internal iliac artery, inferior epigastric artery, or gonadal vein are commonly used for interposition grafts, we chose the SMA due to its larger luminal diameter, which closely matched that of the main renal artery [3–6]. The SMA also offered better structural integrity compared to vein grafts, which have thinner walls and are more prone to occlusion, aneurysms, and rupture [7]. Additionally, graft selection with a vessel of similar tissue composition to the original vessel provides better outcomes [7].

For vascular reconstruction technique, we used end-to-side anastomosis to attach the SMA interposition graft to the external iliac artery. End-to-side anastomosis offers advantages such as better vessel patency at 6 months and reduced cold ischemia time [6, 8, 9]. Additionally, it offers flexibility in vessel lumen diameter, which is important in cases with vessel size mismatch, as it minimizes flow turbulence at the anastomosis site and reduces the risk of thrombosis [6, 8, 9].

Future studies with larger cohorts are warranted to evaluate the long-term patient outcomes, durability of the SMA graft, and graft survival beyond 12 months for the use of SMA as an arterial interposition graft in kidney transplantation.
